# Supplementary material for: Longitudinal changes of ADHD symptoms in association with white matter microstructure: A tract-specific fixel-based analysis
Source: Neuroimage Clin. 2022 May 24;35:103057. doi: 10.1016/j.nicl.2022.103057 (PMC9144034; doi:10.1016/j.nicl.2022.103057)
Supplement: Supplementary data 1 [file mmc1.docx]

# 8. Supplementary material

| **Search space** | **Dependent variable**  **Fixel-based metric** | **Contrast** | **Independent variable**  **Symptom score change**  **(Δ = W2 – W1)** | ***t*_max_** | **Std. effect** | ***P*_FWE_** |
| --- | --- | --- | --- | --- | --- | --- |
| **Left corticospinal tract** | **Fiber density** | ( + ) | combined | 0.858 | 0.011 | 0.165 |
|  |  | ( – ) | combined | 0.917 | 0.012 | 0.104 |
|  |  | ( + ) | HI | 0.949 | 0.038 | 0.225 |
|  |  | **( – )** | **HI** | **1.092** | **0.044** | **0.016*** |
|  | Fiber cross-section | ( + ) | combined | 1.846 | 0.023 | 0.591 |
|  |  | ( – ) | combined | 2.264 | 0.029 | 0.343 |
|  |  | ( + ) | HI | 1.909 | 0.076 | 0.627 |
|  |  | ( – ) | HI | 2.189 | 0.087 | 0.536 |
|  | Fiber density and cross-section | ( + ) | combined | 0.969 | 0.012 | 0.280 |
|  |  | ( – ) | combined | 0.828 | 0.011 | 0.439 |
|  |  | ( + ) | HI | 1.051 | 0.042 | 0.294 |
|  |  | ( – ) | HI | 0.948 | 0.038 | 0.083 |
| Left superior longitudinal fasciculus | Fiber density | ( + ) | combined | 2.238 | 0.028 | 0.548 |
|  |  | ( – ) | combined | 1.462 | 0.019 | 0.327 |
|  | Fiber cross-section | ( + ) | combined | 2.627 | 0.033 | 0.306 |
|  |  | ( – ) | combined | 3.121 | 0.040 | 0.121 |
|  | Fiber density and cross-section | ( + ) | combined | 2.483 | 0.032 | 0.127 |
|  |  | ( – ) | combined | 1.351 | 0.017 | 0.598 |

## **Table S1.** Results from the tract-specific fixel-based analysis of W1 to W2 in lCST and lSLF.

| **Search space** | **Dependent variable**  **Fixel-based metric** | **Contrast** | **Independent variable**  **Symptom score change**  **(Δ = W3 – W2)** | ***t*_max_** | **Std. effect** | ***P*_FWE_** |
| --- | --- | --- | --- | --- | --- | --- |
| **Left corticospinal tract** | Fiber density | ( + ) | combined | 1.288 | 0.017 | 0.612 |
|  |  | ( – ) | combined | 1.687 | 0.023 | 0.156 |
|  |  | ( + ) | HI | 1.344 | 0.036 | 0.636 |
|  |  | ( – ) | HI | 1.761 | 0.048 | 0.200 |
|  | **Fiber cross-section** | ( + ) | combined | 0.916 | 0.012 | 0.896 |
|  |  | **( – )** | **combined** | **3.775** | **0.051** | **0.019*** |
|  |  | ( + ) | HI | 0.911 | 0.025 | 0.960 |
|  |  | ( – ) | HI | 3.575 | 0.097 | 0.058 |
|  | Fiber density and cross-section | ( + ) | combined | 1.122 | 0.015 | 0.821 |
|  |  | ( – ) | combined | 1.776 | 0.024 | 0.093 |
|  |  | ( + ) | HI | 1.190 | 0.032 | 0.908 |
|  |  | ( – ) | HI | 1.800 | 0.049 | 0.141 |
| Left superior longitudinal fasciculus | Fiber density | ( + ) | combined | 2.062 | 0.028 | 0.675 |
|  |  | ( – ) | combined | 1.572 | 0.021 | 0.978 |
|  | Fiber cross-section | ( + ) | combined | 1.450 | 0.019 | 0.940 |
|  |  | ( – ) | combined | 2.863 | 0.038 | 0.259 |
|  | Fiber density and cross-section | ( + ) | combined | 1.928 | 0.026 | 0.639 |
|  |  | ( – ) | combined | 1.823 | 0.024 | 0.835 |

## **Table S2.** Results from the tract-specific fixel-based analysis of W2 to W3 in lCST and lSLF.

| **Search space** | **Dependent variable**  **Fixel-based metric** | **Contrast** | **Independent variable**  **CAARS score**  **(W3)** | ***t*_max_** | **Std. effect** | ***P*_FWE_** |
| --- | --- | --- | --- | --- | --- | --- |
| Whole brain | Fiber density | ( + ) | combined | 3.011 | 0.045 | 0.562 |
|  |  | ( – ) | combined | 2.739 | 0.041 | 0.858 |
|  |  | ( + ) | HI | 3.195 | 0.082 | 0.501 |
|  |  | ( – ) | HI | 2.640 | 0.068 | 0.835 |
|  |  | ( + ) | IA | 3.120 | 0.095 | 0.053 |
|  |  | ( – ) | IA | 2.862 | 0.087 | 0.757 |
|  | Fiber cross-section | ( + ) | combined | 2.775 | 0.042 | 0.835 |
|  |  | ( – ) | combined | 4.446 | 0.067 | 0.261 |
|  |  | ( + ) | HI | 2.172 | 0.056 | 0.892 |
|  |  | ( – ) | HI | 4.218 | 0.109 | 0.173 |
|  |  | ( + ) | IA | 3.721 | 0.113 | 0.700 |
|  |  | ( – ) | IA | 3.749 | 0.114 | 0.362 |
|  | Fiber density and cross-section | ( + ) | combined | 3.027 | 0.046 | 0.678 |
|  |  | ( – ) | combined | 2.847 | 0.043 | 0.778 |
|  |  | ( + ) | HI | 3.111 | 0.080 | 0.864 |
|  |  | ( – ) | HI | 2.774 | 0.071 | 0.827 |
|  |  | ( + ) | IA | 3.166 | 0.096 | 0.119 |
|  |  | ( – ) | IA | 2.840 | 0.086 | 0.716 |
| Right cingulum bundle | Fiber density | ( + ) | HI | 0.047 | 1.841 | 0.410 |
|  |  | ( – ) | HI | 0.043 | 1.678 | 0.315 |
|  | Fiber cross-section | ( + ) | HI | 0.763 | 0.020 | 0.980 |
|  |  | ( – ) | HI | 3.806 | 0.098 | 0.063 |
|  | Fiber density and cross-section | ( + ) | HI | 1.653 | 0.043 | 0.688 |
|  |  | ( – ) | HI | 1.764 | 0.045 | 0.172 |

## **Table S3.** Cross-sectional association between white matter and symptoms at W3 only; results from a global and tract-specific fixel-based analysis. Because we previously found global and tract-specific cross-sectional fractional anisotropy associations in the whole brain and in the rCG in an overlapping W1 sample, we asked whether the same cross-sectional association still existed specifically at W3 in only individuals with a history of ADHD diagnosis (Damatac et al., 2020). Thus, as a secondary cross-sectional analysis in W3 only, we tested for an effect of CAARS score (IA, HI, and combined) on each of the three fixel metrics at the level of the whole brain, as well as rCG only. We predicted that symptom severity would be negatively associated with FD and FC in the whole brain and rCG at the last wave. We used age, sex, and head motion (framewise displacement) as covariates: fixel metric ~ score + age + sex + head motion. None of the global or right cingulum (rCG) WM microstructure metrics were significantly associated with HI, IA, or combined CAARS score cross-sectionally. All general linear models showed no significant effects of CAARS score on any fixel-based metric. Our inability to replicate our previous cross-sectional tractography findings may stem from the different DWI analysis methods used. Also, W3 included a comparatively smaller number of affected-only individuals, which reduced our statistical power to detect smaller cross-sectional effects in the rCG.

## **Figure S1.** Our pre-processing, quality control, and fixel-based analysis pipeline was based on the recommended protocol from the makers of MRtrix3. More details for each step can be found at <https://mrtrix.readthedocs.io/en/latest/fixel_based_analysis/mt_fibre_density_cross-section.html>. DWI data from W2 and from W3 were each processed separately.

1. (Veraart et al., 2016)
2. (Kellner et al., 2016)
3. (Andersson and Sotiropoulos, 2016)
4. (Smith et al., 2004)
5. (Zwiers, 2010)
6. (Tustison et al., 2010)
7. (Dhollander et al., 2016)
8. (Tournier et al., 2019)
9. (Jeurissen et al., 2014)
10. (Tournier et al., 2007)
11. (D. Raffelt et al., 2017)
12. (Raffelt et al., 2011)
13. (Raffelt et al., 2012)
14. (Smith et al., 2013)
15. (Raffelt et al., 2015)
16. (Tournier et al., 2010)
17. (Wasserthal et al., 2018)
18. (Winkler et al., 2014)
19. (Winkler et al., 2015)

## **Figure S2.** Correlation scatterplots of baseline against follow-up scores colored by dimension with 95% confidence intervals and Pearson correlation coefficients reported for each. **Top row:** Wave 1 associations with Wave 2 scores. **Bottom row:** Wave 2 associations with Wave 3 scores. **Left column**: Hyperactivity-impulsivity dimension scores. **Center column:** Inattention dimension scores. **Right column:** Combined scores (calculated as the sum of hyperactivity-impulsivity and inattention). Darker colored points indicate individuals with overlapping score data.


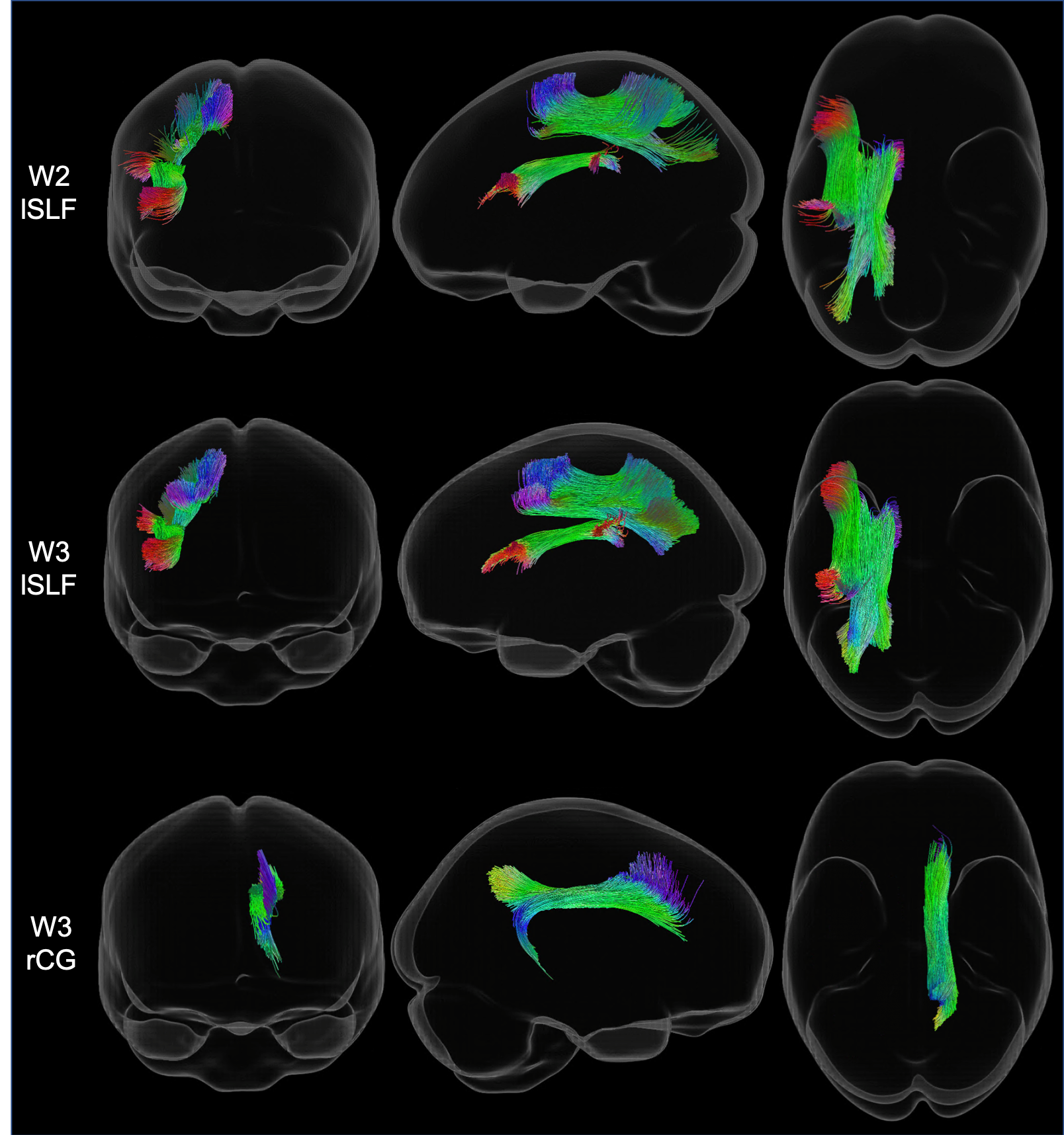


## **Figure S3.** Tract-specific region-of-interest masks of the left superior longitudinal fasciculus (lSLF) at Wave 2 and Wave 3, and the right cingulum bundle (rCG) at Wave 3 colored by direction (red: left-right, green: anterior-posterior, blue: inferior-superior). Coronal (left column), sagittal (middle column), and axial (right column) views of the tract reconstructions from TractSeg (applied to the fiber orientation distribution templates) and displayed in glass brains for visualization.


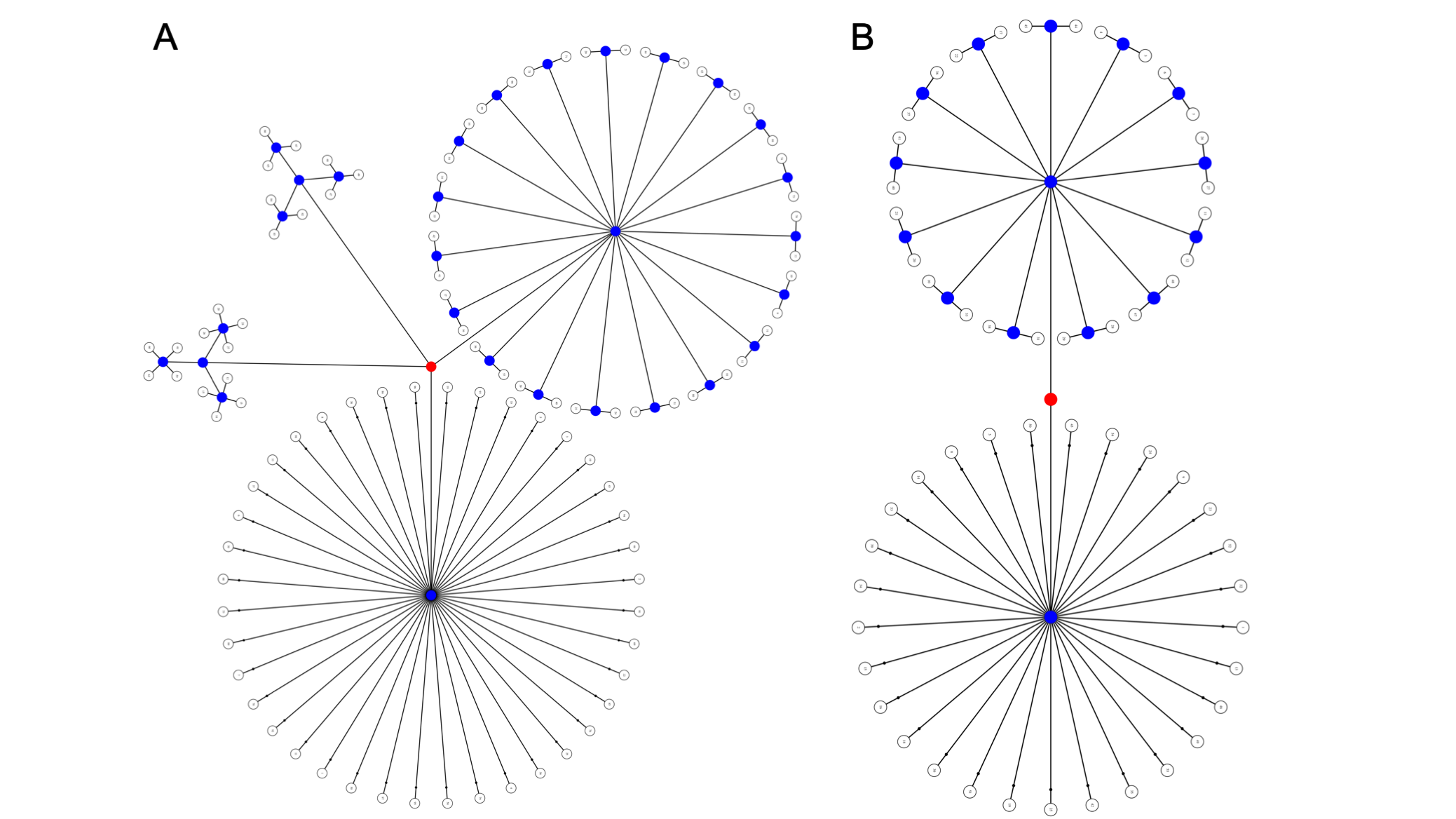


## **Figure S4.** Visualization of the exchangeability block structure of (A) Wave 2 and (B) Wave 3, each represented in permutation trees. Each white dot is one individual and each group of white dots represents families of a specific size. At each permutation, branches beginning at blue dots can be permuted, while those beginning in red dots cannot. These permutation sets were generated with FSL PALM and used in connectivity-based fixel enhancement analysis to control for related siblings in each sample.


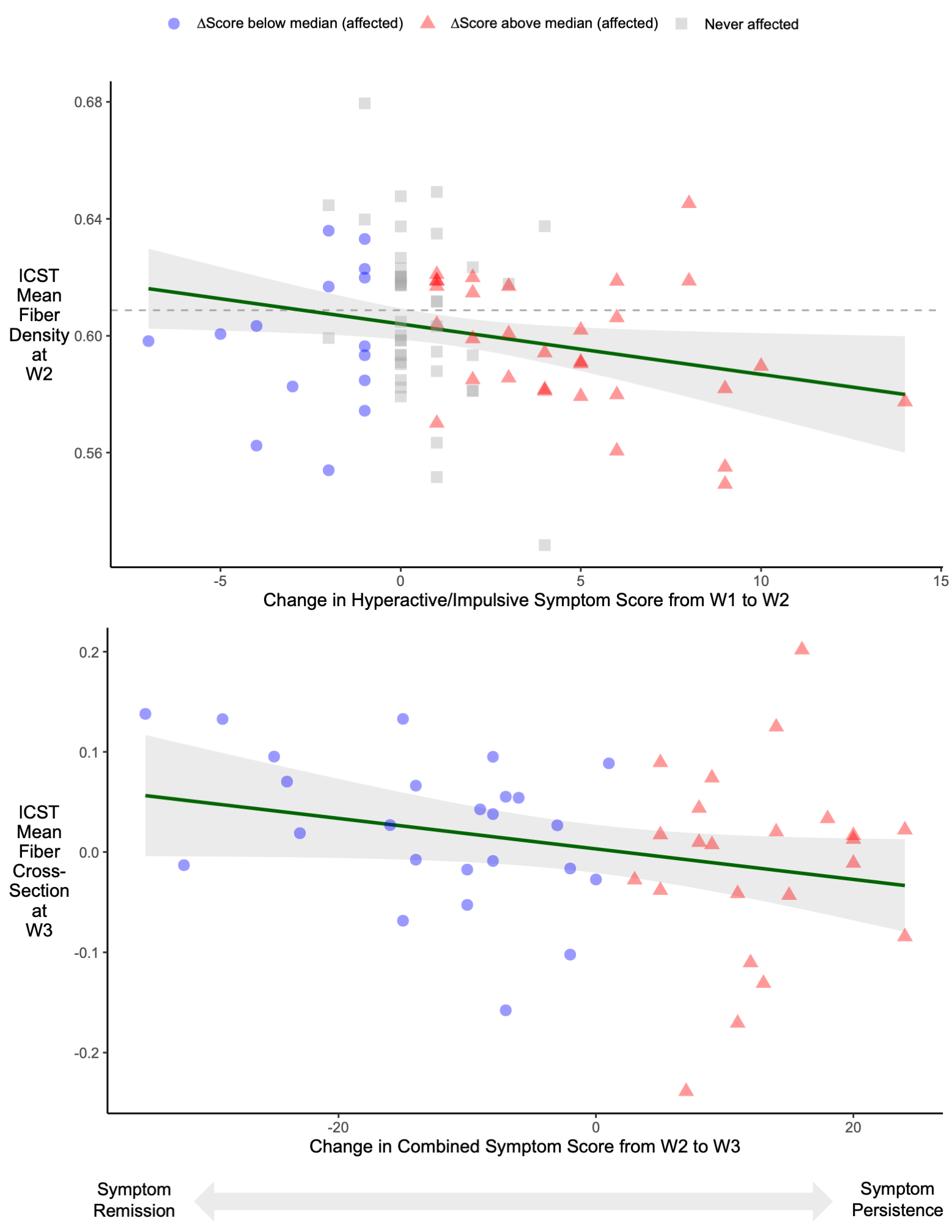


## **Figure S5.** Graphs illustrating the relationship between left corticospinal tract (**Top**) mean fiber density values and change in hyperactive/impulsive symptom score from Wave 1 to Wave 2, and (**Bottom**) mean fiber cross-section values and change in combined symptom score from Wave 2 to Wave 3. Both plots include solid green regression lines with 95% confidence intervals. For reference, the mean fiber density at Wave 2 for unaffected participants is marked by a dashed gray line. Change in symptom score was calculated as: ΔScore = score _follow-up_ – score _baseline_. Participants are shown as individual points, differentiated according to whether their change in symptom score was above or below the median change in score.

## **Figure S6.** Participants from all waves pooled together (never affected are in gray triangles, and affected are circles colored according to wave in red [W1], blue [W2], and green [W3]) and plotted by age against symptom score in dimensions HI (**Top**) and IA (**Bottom**). Wave 3 included less participants and only those with a history of ADHD; scores were obtained with CAARS instead of CPRS.


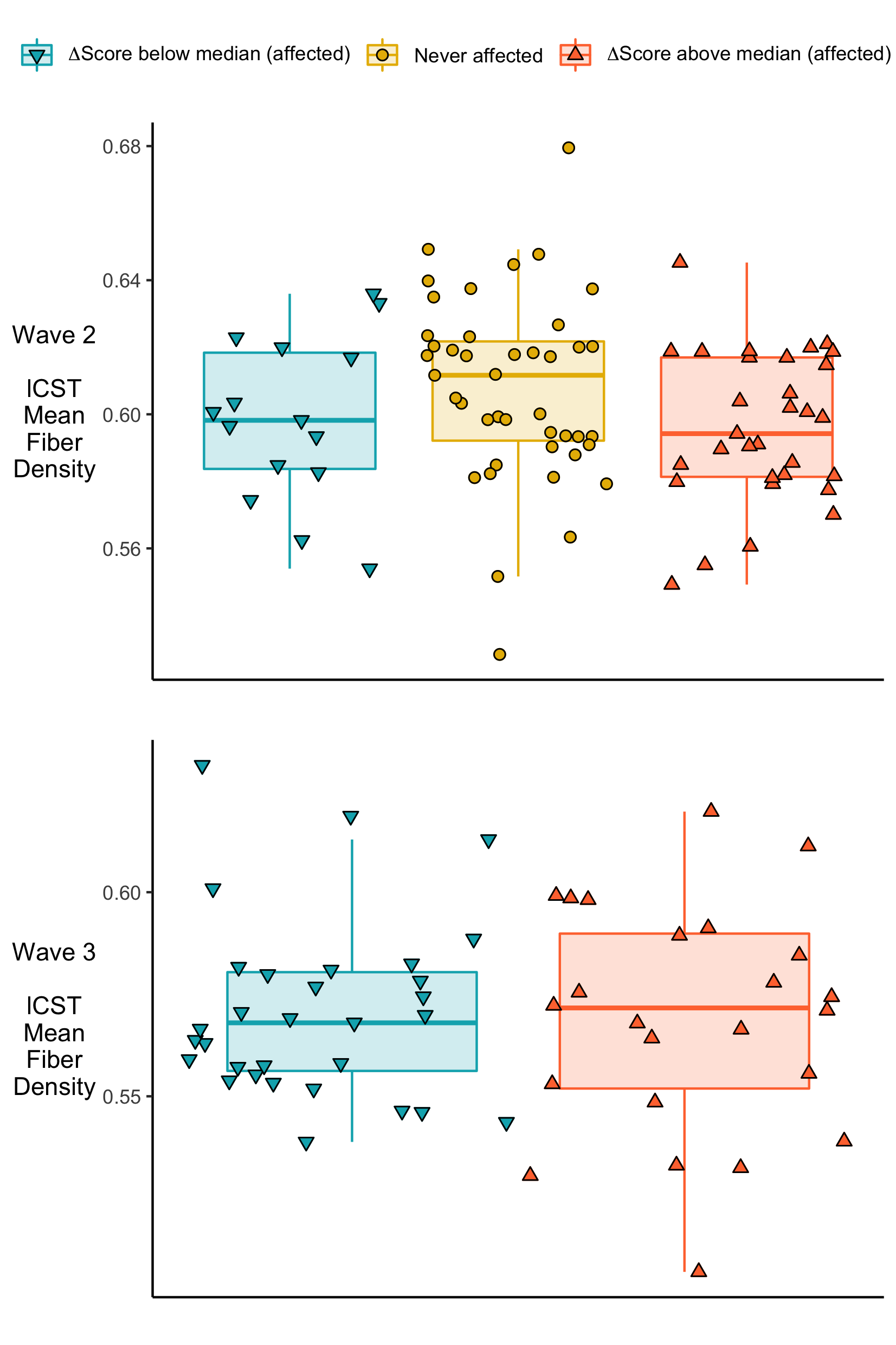


## **Figure S7.** Jittered boxplots of mean fiber density (FD) within the left corticospinal tract (lCST) fixel masks at Wave 2 (**Top**) and Wave 3 (**Bottom**). Participants are represented by individual points and grouped according to whether their change in symptom score was above or below the median change in score (and unaffected from Wave 1 to Wave 2). Change in symptom score was calculated as: ΔScore = score _follow-up_ – score _baseline_. Wave 2 diffusion-weighted data was acquired at b=1000, while Wave 3 was acquired at b=2500. While FD estimates are generally negatively associated with diffusion weighting, diffusion-weighting in itself did not confound our effect of interest since the effect is balanced across both waves and b-values.
